# Supplementary material for: Cholangiocarcinoma Malignant Traits Are Promoted by Schwann Cells through TGFβ Signaling in a Model of Perineural Invasion
Source: Cells. 2024 Feb 20;13(5):366. doi: 10.3390/cells13050366 (PMC10930666; doi:10.3390/cells13050366)
Supplement: Supplementary file 1 [file cells-13-00366-s001.zip › cells-2784463-supplementary.pdf]

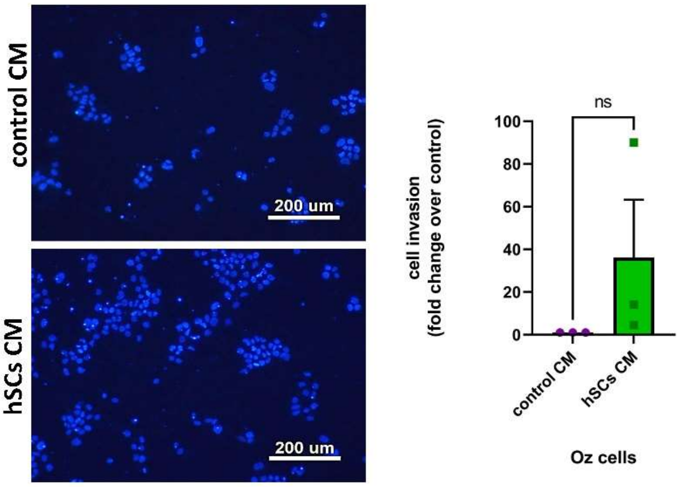


**Figure S1**.Oz cells exposed to hSC conditioned medium displayed a trend (yet statistically unsignificant) of increased Matrigel invasiveness. Micrographs and related diagrams show the increase of Matrigel invasion in Oz cells treated with hSCs CM respect to control CM. Student's t-test: p= 0,13. N=3

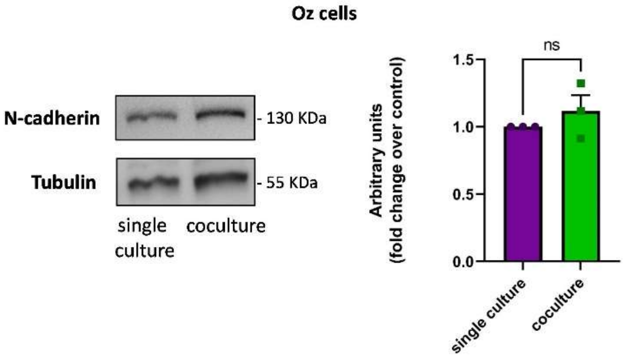


**Figure S2.** Western blot analyses of N-cadherin expression in Oz cells cocultured with hSCs. The figure shows an upregulation trend (yet statistically unsignificant) of mesenchymal marker N-cadherin in the coculture sample. The graph depicts the result of densitometric analyses. Student's t-test: p = 0.19; N=3.

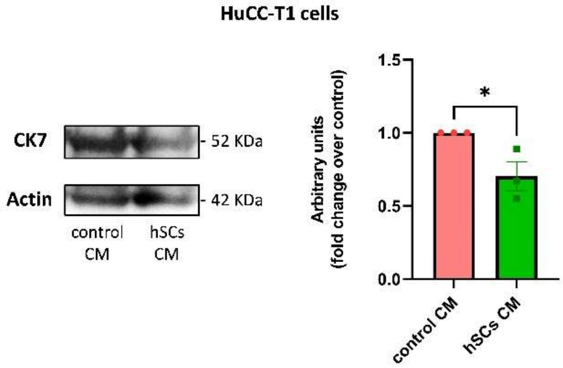


**Figure S3.** SCs CM-induced downregulation of cytokeratin 7 (CK7) in HuCC-T1 cells. Western blot analysis of CK7 expression in HuCC-T1 cells exposed to hSCs CM. HuCC-T1 cells treated with hSCs CM exhibited lower levels of cytokeratin 7 (CK7), compared to the control group. The graph depicts the result of densitometric analysis. Student's t-test: *p≤ 0.05. N=3.

| | Accession | Protein escription | Abundance Ratio: coculture / single culture | Abundance Ratio Adj. P-Value: coculture / single culture | number of detections in HuCC-T1 single culture | number of detections in HuCC-T1/hSCs cocultures |
|---|---|---|---|---|---|---|
| 1 | O14576-1 | cytoplasmic dynein 1 intermediate chain 1 | 100 | 6,50E-16 | 0 | 4 |
| 2 | O15239 | NADH dehydrogenase [ubiquinone] 1 alpha subcomplex subunit 1 | 100 | 6,50E-16 | 0 | 4 |
| 3 | P07093-1 | Glia-derived nexin | 100 | 6,50E-16 | 0 | 3 |
| 4 | Q02388-1 | Collagen alpha-1(VII) chain | 100 | 6,50E-16 | 0 | 3 |
| 5 | Q68CP9-1 | AT-rich interactive domain-containing protein 2 | 2,40 | 4,32E-02 | 4 | 4 |
| 6 | P51991-2 | Isoform 2 of Heterogeneous nuclear ribonucleoprotein A3 | 2,19 | 1,81E-09 | 4 | 4 |
| 7 | P11171-1 | protein 4.1 | 2,16 | 3,55E-06 | 4 | 4 |
| 8 | P62306 | Small nuclear ribonucleoprotein F | 2,05 | 2,18E-04 | 4 | 4 |
| 9 | Q6IQ49-1 | Protein SDE2 homolog | 2,63 | 4,70E-02 | 1 | 3 |
| 10 | Q96KC8 | DnaJ homolog subfamily C member 1 | 2,34 | 1,10E-02 | 3 | 3 |
| 1 | Q15828 | Cystatin-M | 0,01 | 6,50E-16 | 3 | 0 |
| 2 | Q9UKQ2-1 | Disintegrin and metalloproteinase domain-containing protein 28 | 0,01 | 6,50E-16 | 3 | 0 |
| 3 | P67775 | Serine/threonine-protein phosphatase 2A catalytic subunit alpha isoform | 0,05 | 6,50E-16 | 4 | 3 |
| 4 | P13929-1 | Beta-enolase | 0,39 | 8,50E-14 | 4 | 4 |
| 5 | P43308 | Translocon-associated protein subunit beta | 0,41 | 3,58E-05 | 4 | 4 |
| 6 | P53794 | sodium/myo-inositol cotransporter | 0,42 | 1,11E-09 | 4 | 4 |
| 7 | P69905 | Hemoglobin subunit alpha | 0,43 | 6,50E-16 | 4 | 4 |
| 8 | Q9UGU0 | Transcription factor 20 | 0,45 | 1,03E-02 | 4 | 4 |
| 9 | P32456 | Guanylate-binding protein 2 | 0,45 | 1,33E-02 | 4 | 4 |
| 10 | Q9UM47 | Neurogenic locus notch homolog protein 3 | 0,50 | 7,93E-08 | 4 | 4 |

**Table S1.** Proteins differentially regulated in HuCC-T1 cells cocultured with hSCs identified by proteomic analysis. The table shows the complete list of modulated proteins in HuCC-T1/hSCs coculture respect to controls, with the respective accession numbers and p values. Red: upregulated proteins; Blue: downregulated proteins. T-test, Benjamini-corrected. N=4.
